# Supplementary material for: Proteomic Landscape and Deduced Functions of the Cardiac 14-3-3 Protein Interactome
Source: Cells. 2022 Nov 4;11(21):3496. doi: 10.3390/cells11213496 (PMC9654263; doi:10.3390/cells11213496)
Supplement: Supplementary file 1 [file cells-11-03496-s001.zip › Supplementary_Figures.pdf]

Supplementary Figures

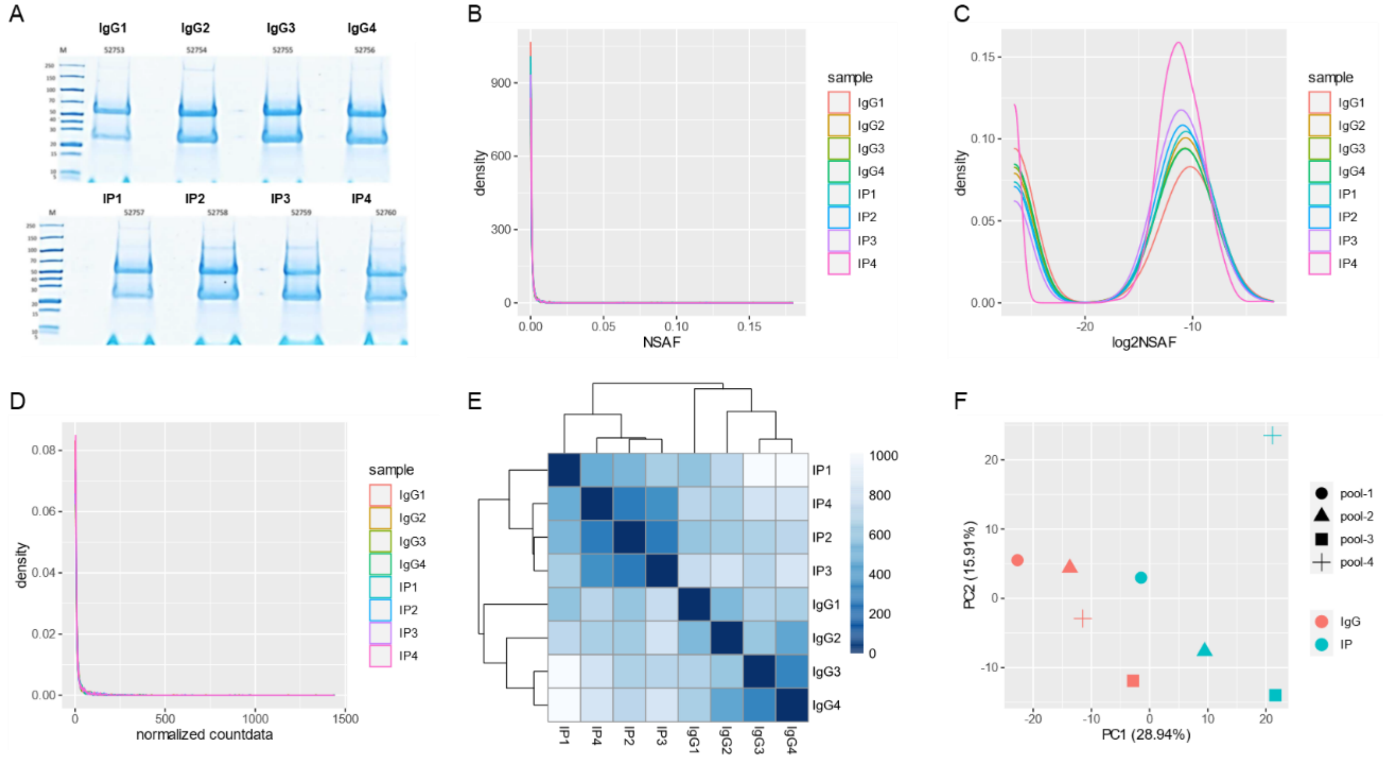

Supplementary Figure S1. Identification of 14-3-3 interacting proteins in mouse heart. A. Image of a coomassie stained gel of sample lysates, four IgG and four IP samples. B-D. Distribution plots of normalized protein counts: (B) NSAF, (C)log2NSAF, and (D) normalized count in R package, countdata. E. Sample distance matrix. The closer two samples are, the deeper color their intersection is. F. PCA plot of all samples.

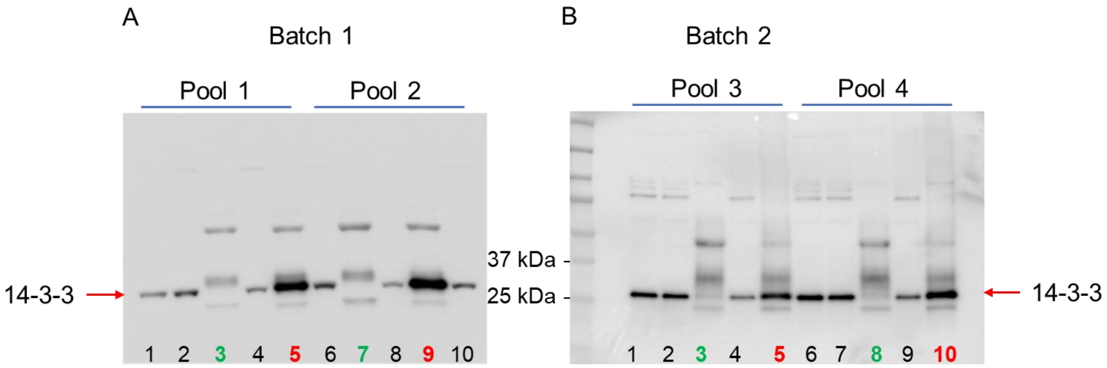

Lanes:
1 – Pool 1 Input
2 – Pool 1 Isotype Ab IP Flow Through
3 – Pool 1 Isotype Ab IP Elution
4 – Pool 1 14-3-3 Ab Flow Through
5 – Pool 1 14-3-3 Ab IP Elution
6 – Pool 2 Isotype Ab IP Flow Through
7 – Pool 2 Isotype Ab IP Elution
8 – Pool 2 14-3-3 Ab Flow Through
9 – Pool 2 14-3-3 Ab IP Elution
10 – Pool 2 Input

Lanes:
1 – Pool 3 Input
2 – Pool 3 Isotype Ab IP Flow Through
3 – Pool 3 Isotype Ab IP Elution
4 – Pool 3 14-3-3 Ab Flow Through
5 – Pool 3 14-3-3 Ab IP Elution
6 – Pool 4 Input
7 – Pool 4 Isotype Ab IP Flow Through
8 – Pool 4 Isotype Ab IP Elution
9 – Pool 4 14-3-3 Ab Flow Through
10 – Pool 4 14-3-3 Ab IP Elution

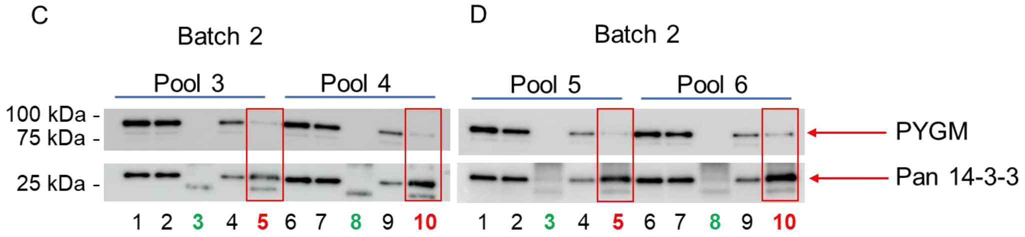

Lanes:
1 – Pool 3 Input
2 – Pool 3 Isotype Ab IP Flow Through
3 – Pool 3 Isotype Ab IP Elution
4 – Pool 3 14-3-3 Ab Flow Through
5 – Pool 3 14-3-3 Ab IP Elution
6 – Pool 4 Input
7 – Pool 4 Isotype Ab IP Flow Through
8 – Pool 4 Isotype Ab IP Elution
9 – Pool 4 14-3-3 Ab Flow Through
10 – Pool 4 14-3-3 Ab IP Elution

Lanes:
1 – Pool 5 Input
2 – Pool 5 Isotype Ab IP Flow Through
3 – Pool 5 Isotype Ab IP Elution
4 – Pool 5 14-3-3 Ab Flow Through
5 – Pool 5 14-3-3 Ab IP Elution
6 – Pool 6 Input
7 – Pool 6 Isotype Ab IP Flow Through
8 – Pool 6 Isotype Ab IP Elution
9 – Pool 6 14-3-3 Ab Flow Through
10 – Pool 6 14-3-3 Ab IP Elution

Supplementary Figure S2. Validation of proteins via western blotting following co-IP against 14-3-3. The co-IP experiment was conducted in two batches while mouse left ventricle tissues were aggregated into six pools. A-B. Western blots with two different antibodies to pan-14-3-3, Santa Cruz Biotechnology, Inc; Cat. No. sc-133233 (A) and ThermoFisher Scientific; Cat. No. 51-0700 (B). C. Western blots with antibodies to pan-14-3-3 (ThermoFisher Scientific; Cat. No. 51-0700) and PYGM (Proteintech, Rosemont, IL; Cat. No. 19716-1-AP). Sampling orders in each lane were shown below blot.

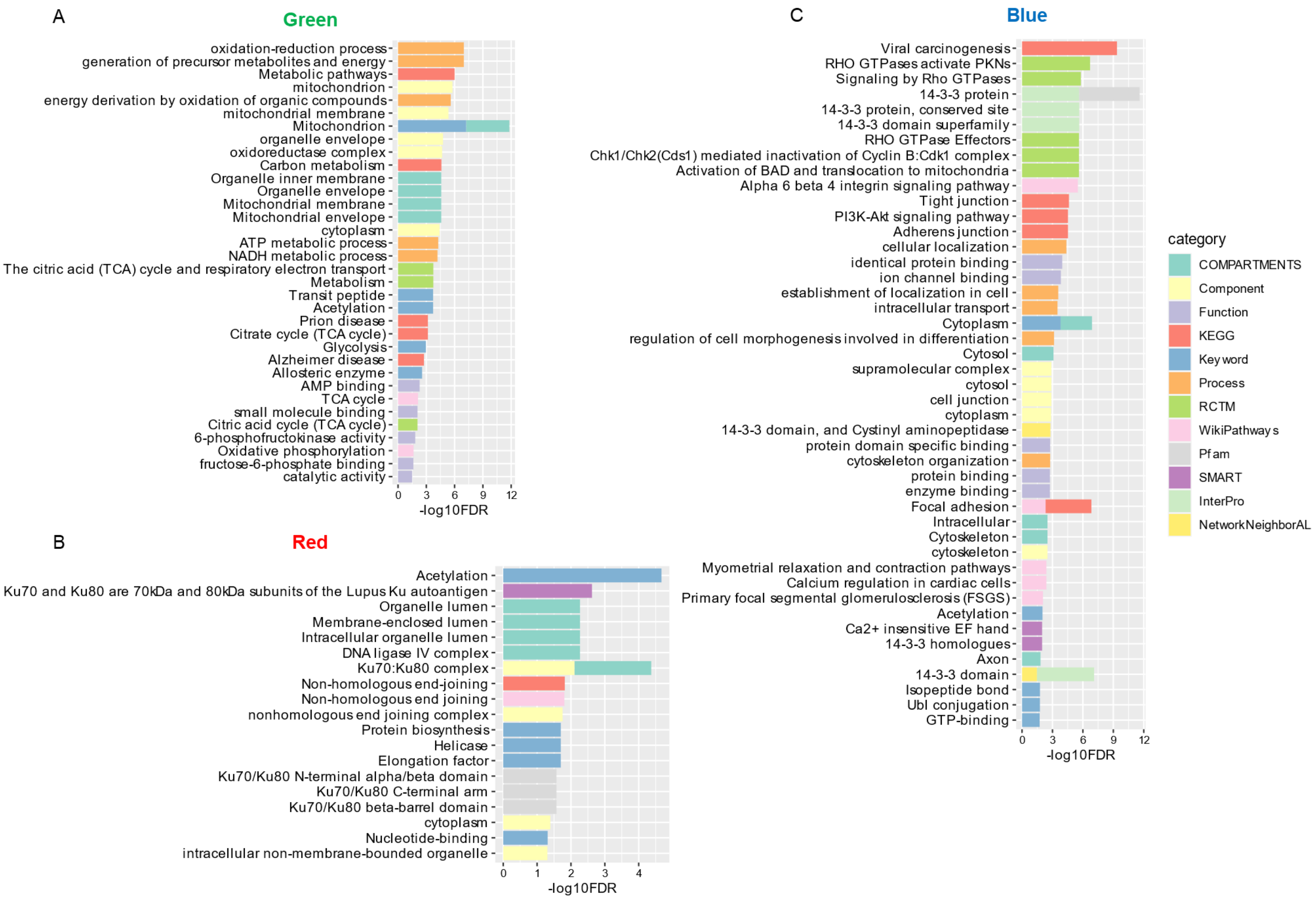

Supplementary Figure S3. Features of the three clusters in Figure 4A. The features of the three clusters were analyzed with the knowledgebase in 12 public databases. The 12 categories are: COMPARTMENTS from Subcellular localization (COMPARTMENTS) database, Component from Cellular Component (Gene Ontology) database, Function from Molecular Function (Gene Ontology) database, InterPro from Protein Domain and Features (InterPro) database, KEGG from Kyoto Encyclopedia of Genes and Genomes pathway (KEGG) database, Key word from Annotated Keywaords (Uniprot) database, NetworkNeighborAL from local network cluster (STRING) database, Pfam from Protein Domains (Pfam) database, Process from Biological Process (Gene Ontology) database, RCTM from Reactome Pathways database, SMART from Protein Domains (SMART) database, and WikiPathways from WikiPathways database. If there were more than five terms in one method with FDR < 0.05, only the top five were remained for display; otherwise, all terms in the method were remained. All terms enriched from the same cluster were displayed in the same bar plot. The bar representing terms in two methods were in two colors.

A BP

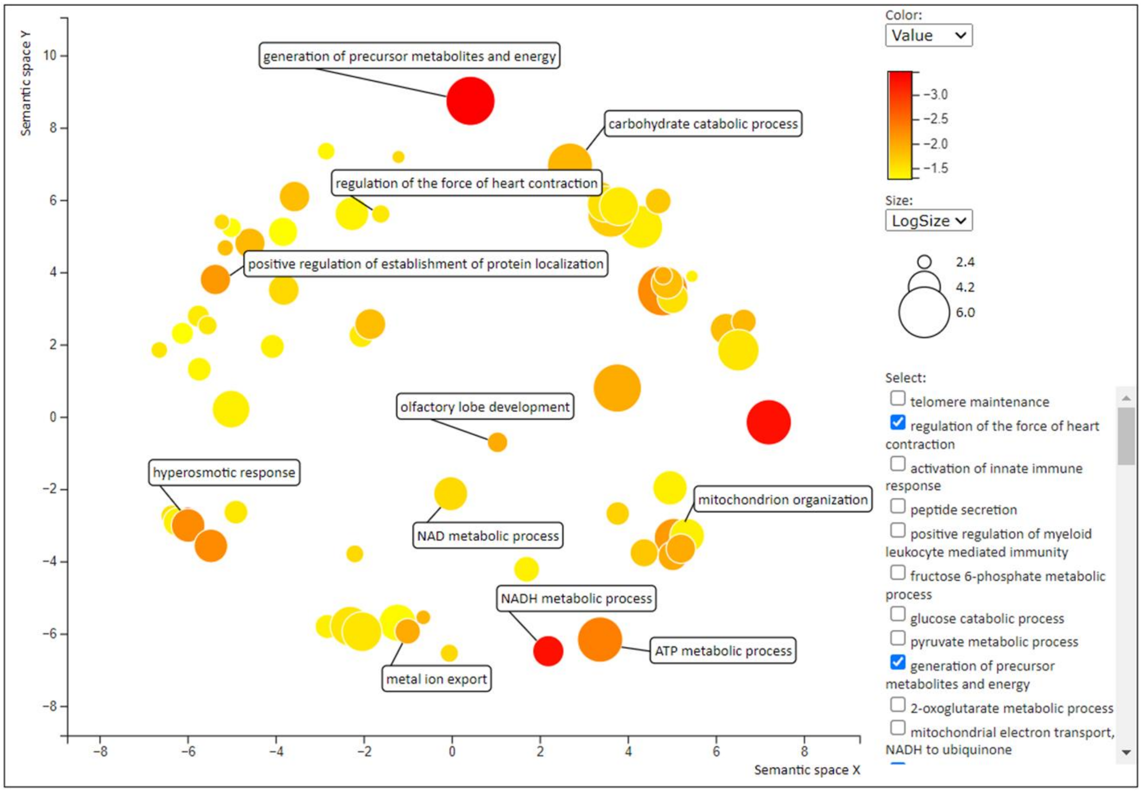

Semantic space Y
Semantic space X
generation of precursor metabolites and energy
carbohydrate catabolic process
regulation of the force of heart contraction
positive regulation of establishment of protein localization
olfactory lobe development
hyperosmotic response
NAD metabolic process
mitochondrion organization
NADH metabolic process
ATP metabolic process
metal ion export
Color:
Value
Size:
LogSize
Select:
telomere maintenance
regulation of the force of heart contraction
activation of innate immune response
peptide secretion
positive regulation of myeloid leukocyte mediated immunity
fructose 6-phosphate metabolic process
glucose catabolic process
pyruvate metabolic process
generation of precursor metabolites and energy
2-oxoglutarate metabolic process
mitochondrial electron transport, NADH to ubiquinone

B CC

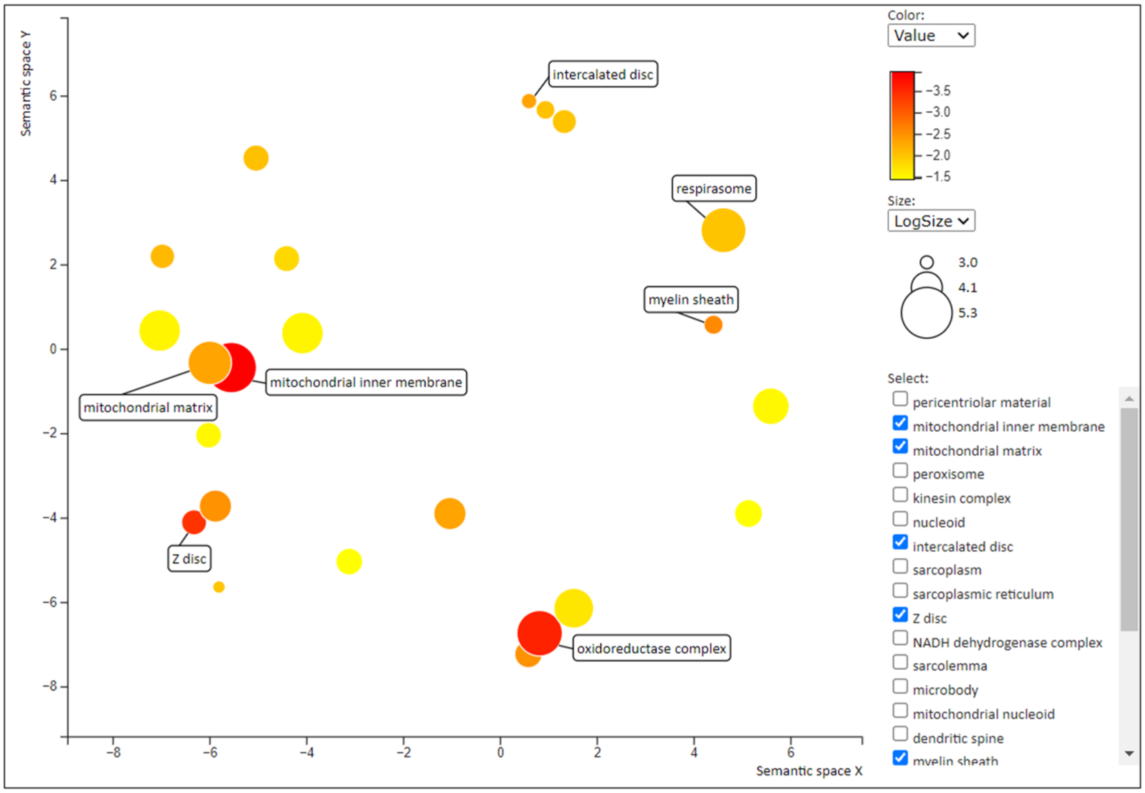

Semantic space Y
Semantic space X
intercalated disc
respirasome
myelin sheath
mitochondrial inner membrane
mitochondrial matrix
Z disc
oxidoreductase complex
Color:
Value
Size:
LogSize
Select:
pericentriolar material
mitochondrial inner membrane
mitochondrial matrix
peroxisome
kinesin complex
nucleoid
intercalated disc
sarcoplasm
sarcoplasmic reticulum
Z disc
NADH dehydrogenase complex
sarcolemma
microbody
mitochondrial nucleoid
dendritic spine
myelin sheath

C MF

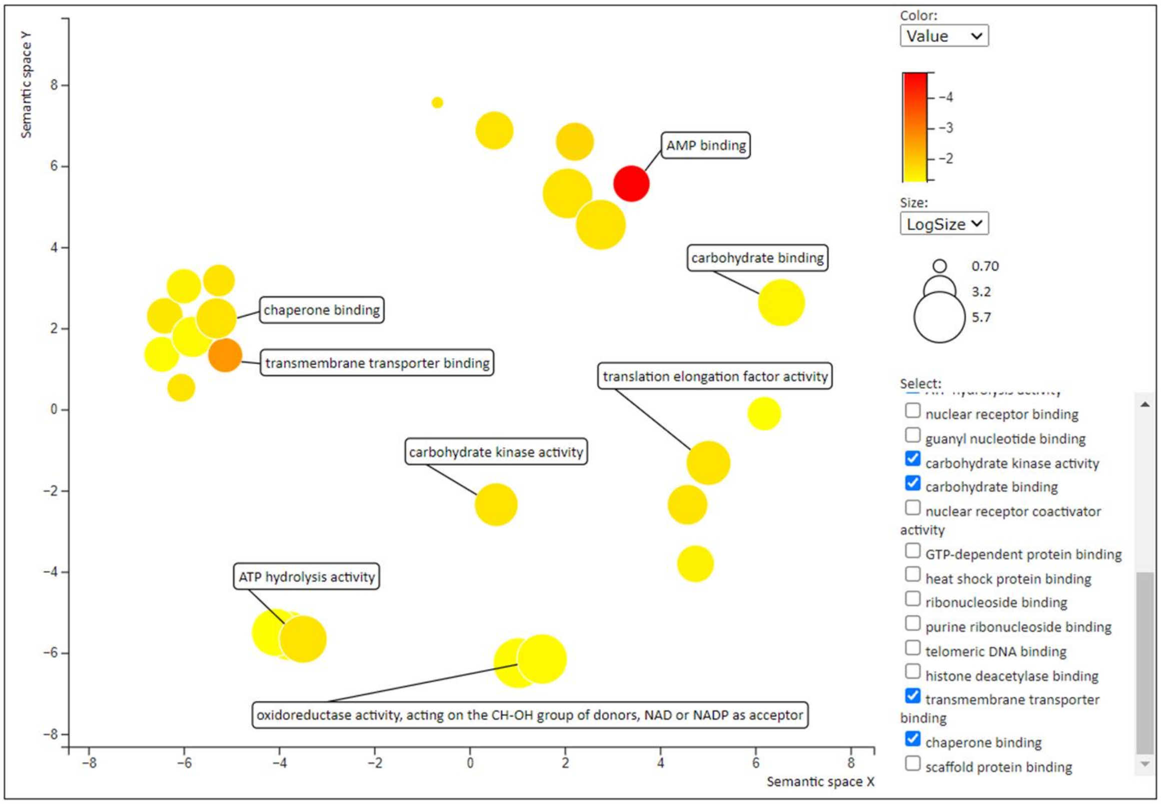

Supplementary Figure S4. Visualization of GO terms after redundancy reduction in REVIGO. GO terms obtained in clusterProfiler were imported into REVIGO to reduce redundancy and then visualized in bubble chart. The bubbles' x and y coordinates (semantic space x, y) were derived by applying multidimensional scaling to a matrix of the GO terms' semantic similarities. Consequently, their closeness on the plot should closely reflect their closeness in the GO graph structure i.e., the semantic similarity. Bubble color ranging from yellow to red indicates log10(p-value) of enrichment and the bubble whose color is closer to red is more significant; bubble size indicates the frequency of the GO term in the underlying GOA database (bubbles of more general terms are larger). The terms of several enrichments with great significance are displayed next to the bubble. Three categories were BP (Biological Process, A), CC (Cellular Component, B) and MF (Molecular Function, C).
